# An Amperometric Enzyme–Nanozyme Biosensor for Glucose Detection

**DOI:** 10.3390/bios15080545

**Published:** 2025-08-19

**Authors:** Asta Kausaite-Minkstimiene, Aiste Krikstaponyte, Nataliya Stasyuk, Galina Gayda, Almira Ramanaviciene

**Affiliations:** 1 NanoTechnas–Center of Nanotechnology and Materials Science, Faculty of Chemistry and Geosciences, Vilnius University, Naugarduko st. 24, LT-03225 Vilnius, Lithuania; aiste.krikstaponyte@chgf.vu.lt; 2 Department of Analytical Biotechnology, Institute of Cell Biology National Academy of Sciences of Ukraine (ICB NASU), Dragomanova Str. 14/16, 79005 Lviv, Ukraine; stasukne@nas.gov.ua (N.S.); galina.gayda@nas.gov.ua (G.G.)

**Keywords:** glucose biosensor, glucose oxidase, nanozyme, artificial peroxidase

## Abstract

Amperometric biosensors, due to their high sensitivity, fast response time, low cost, simple control, miniaturization capabilities, and other advantages, are receiving significant attention in the field of medical diagnostics, especially in monitoring blood glucose levels in diabetic patients. In this study, an amperometric glucose biosensor based on immobilized enzyme glucose oxidase (GOx) and bimetallic platinum cobalt (PtCo) nanoparticles was developed. The PtCo nanoparticles, deposited on a graphite rod electrode, exhibited peroxidase-like catalytic properties and were able to electrocatalyze the reduction of H_2_O_2_. After immobilization of the GOx, an amperometric signal generated by the biosensor was directly proportional to the glucose concentration in the range of 0.04–2.18 mM. The biosensor demonstrated a sensitivity of 19.38 μA mM^−1^ cm^−2^, with a detection limit of 0.021 mM and a quantification limit of 0.064 mM. In addition to this analytical performance, the biosensor exhibited excellent repeatability (relative standard deviation (RSD) was 4.90%); operational and storage stability, retaining 98.93% and 95.33% of its initial response after 26 cycles of glucose detection and over a 14-day period, respectively; and anti-interference ability against electroactive species, as well as exceptional selectivity for glucose and satisfactory reproducibility (RSD 8.90%). Additionally, the biosensor was able to detect glucose levels in blood serum with a high accuracy (RSD 5.89%), indicating potential suitability for glucose determination in real samples.

## 1. Introduction

Although the first enzyme-based electrode for glucose detection was published by Clark and Lyons as early as 1962 [1], the field of glucose biosensor development continues to grow significantly to this day. This is not surprising, because more than 800 million people around the world have diabetes [2], a chronic metabolic disorder characterized by elevated blood glucose levels resulting from insufficient insulin production or ineffective insulin utilization [3]. Meanwhile, glucose biosensors are a very important analytical tool in diagnosing diabetes. Moreover, they also play a crucial role in monitoring fluctuations in glucose levels and making informed decisions regarding medication dosages, dietary choices, and lifestyle changes, thereby reducing the risk of diabetes-related complications. Amperometric glucose biosensors based on GOx are among the most commonly developed biosensors for glucose detection. They have attracted considerable attention due to their simple design, low production cost, user-friendly nature, potential for miniaturization and portability, high sensitivity and selectivity for glucose, and short response time [4]. However, despite being an invaluable analytical tool for efficient and effective glucose determination, these biosensors also have drawbacks, such as interference from other electroactive compounds [5], which makes glucose measurements inaccurate, or limited long-term stability [6], which eventually degrades the performance of the biosensor. The desire to overcome these shortcomings and improve the performance of biosensors encourages scientists to search for new enzyme immobilization techniques and efficient electron transfer mediators, as well as to use various new innovative materials. Among these materials, nanozymes, synthetic materials that mimic the catalytic functions of natural enzymes [7], open up new opportunities in the field of biosensor development by significantly improving their performance. Compared with natural enzymes, they have several advantages, such as simpler production processes, lower costs, longer shelf life, and outstanding stability under various environmental conditions [8].

Nanozymes with different chemical compositions, including metal nanoparticles, metal oxides, carbon nanotubes, and graphene, have been used to develop biosensors. However, metal nanoparticle-based nanozymes composed of one or more metals are perhaps the most commonly used due to their reliable and stable catalytic properties [9]. Nanozymes of this type often exhibit strong enzyme-like catalytic activity because they can easily donate or accept electrons during redox reactions, which is crucial for mimicking the natural functions of oxidases, peroxidases (PO), or catalases. Metal nanoparticle-based nanozymes with PO-like catalytic properties (PO-MNZs) have been shown to be highly effective in H_2_O_2_ chemosensors [10,11,12,13]. Furthermore, as H_2_O_2_ detection is an advantageous way to couple oxidase-catalyzed and electrochemical reactions, PO-MNZs have also proven to be promising materials for developing oxidase-based amperometric biosensors. This enzyme–nanozyme biosensor design enables the selective detection of an analyte through the PO-MNZ-catalyzed electroreduction of H_2_O_2_, which is formed during an enzymatic reaction in the presence of dissolved O_2_ [14]. It has been observed that amperometric biosensors based on PO-MNZs and oxidases are a promising alternative to traditional enzyme-based biosensors due to their higher stability, sensitivity, and cost-effectiveness. For example, amperometric biosensors with high sensitivity for ethanol, low limits of detection (LOD), broad linear ranges and satisfactory storage stabilities have been developed using trimetallic AuCePt and FePtAu nanoparticles in combination with alcohol oxidase [15]. Similarly, CeCu, NiPtPd, and CuHCF, combined with L-arginine oxidase, showed excellent detection efficiency for L-arginine, and the developed biosensors exhibited high sensitivity and selectivity, a wide linear range, and good storage stability [16]. Another amperometric biosensor has been developed based on the use of bimetallic CuFe nanoparticles and cholesterol oxidase on a nanoplatinised glassy carbon electrode (GCE). The biosensor exhibited high sensitivity to cholesterol, wide linear range, and satisfactory storage stability, and was able to accurately detect cholesterol in human serum samples [9]. Although amperometric biosensors employing other oxidases have been developed and studied, those based on GOx have garnered the most attention. For instance, an amperometric biosensor based on Cu nanoflowers and GOx deposited on Pt wire electrode exhibited a wide linear range, with good sensitivity and a fast response time [17]. Furthermore, some GOx-PO-MNZ-based glucose biosensors employ unique design strategies, exploiting the properties of other nanomaterials and thereby further improving their performance. For example, an amperometric glucose biosensor based on PtPd nanoparticles, supported by reduced graphene oxide and integrated with GOx, showed excellent glucose detection performance, featuring high sensitivity, low LOD, short response time, and good stability [18]. An amperometric glucose biosensor based on NiFe_2_/ordered mesoporous carbon nanocomposite and GOx-modified GCE also showed good analytical performances in terms of high sensitivity, low LOD, and wide detection range, and showed great promise for application in the detection of glucose [19]. Thus, it can be assumed that the integration of nanozymes with natural enzymes into the construction of amperometric glucose biosensors can help to create more reliable and cost-effective glucose monitoring devices both in vitro and in vivo.

**Figure 1:**
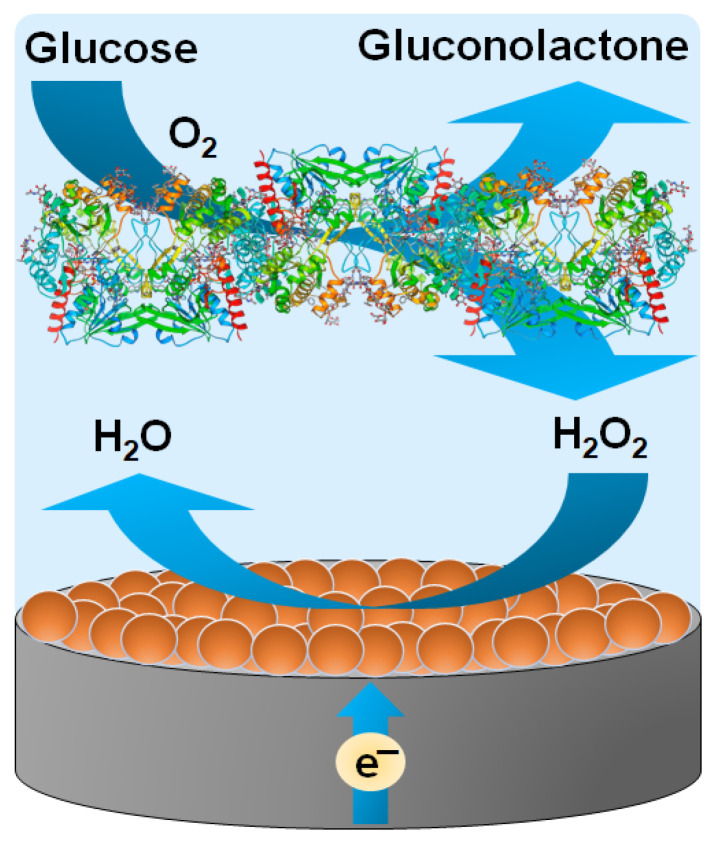
Illustration of the simplified operating principle of the developed amperometric enzyme–nanozyme biosensor.

This work presents the development and investigation of an amperometric GOx-PO-MNZ-based biosensor for the analysis of glucose-containing samples. To develop the biosensor, a graphite rod electrode (GRE) was selected as the working electrode and sequentially modified with PtCo nanoparticles, GOx, and Nafion™ perfluorinated resin using a layer-by-layer deposition approach. The PtCo, deposited on GRE, exhibited PO-like catalytic properties and was able to electrocatalyze the reduction of H_2_O_2_. Thus, the developed biosensor exploited the glucose oxidation reaction catalyzed by immobilized GOx in the presence of dissolved O_2_, along with the ability of PtCo nanoparticles, to catalyze the reduction of enzymatically produced H_2_O_2_ (Figure 1). This combined action of the native enzyme and the nanozyme generated a current that was proportional to the glucose concentration over a certain range. First of all, the conditions of preparation of the working electrode of the biosensor were optimized; then, the main analytical characteristics of the biosensor and the influence of interfering substances were investigated; and, finally, the ability of the biosensor to determine glucose levels in human blood serum samples was verified.

## 2. Materials and Methods

### 2.1. Materials and Reagents

GOx (from *Aspergillus niger*, a lyophilized powder containing 360 U/mg protein), sodium acetate (CH_3_COONa; ≥99%, p.a., ACS, anhydrous), disodium hydrogen phosphate (Na_2_HPO_4_; ≥99%, p.a., ACS, anhydrous), sodium dihydrogen phosphate dodecahydrate (NaH_2_PO_4_ × 12 H_2_O; ≥99%, p.a., ISO), potassium chloride (KCl; ≥99%), acetic acid (CH_3_COOH; 100%, Ph. Eur., extra pure), hydrochloric acid (HCl; 37% fuming, p.a., ACS, ISO), sodium hydroxide (NaOH; ≥99%, beads), D(+)-mannose (C_6_H_12_O_6_; ≥99%), D(+)-xylose (C_6_H_12_O_6_; ≥99%) and D(+)-galactose (C_6_H_12_O_6_; ≥98%) were obtained from Carl Roth GmbH (Karlsruhe, Germany). Human Serum, Type AB, pursed from UAB Labochema LT, Vilnius, Lithuania, Nafion™ perfluorinated resin solution (5 wt.% in lower aliphatic alcohols and water, contains 15–20% water), hydrogen peroxide (H_2_O_2_; 30% (*w*/*w*) in H_2_O, contains stabilizer), acetaminophen (CH_3_CONHC_6_H_4_OH; ≥99.0%), L-ascorbic acid (C_6_H_8_O_6_; 99%), dopamine hydrochloride ((HO)_2_C_6_H_3_CH_2_CH_2_NH_2_ × HCl; ≥98%), uric acid (C_5_H_4_N_4_O_3_; ≥99.0%), chloroplatinic acid hydrate (H_2_PtCl_6_ × H_2_O, ≥99.9% trace metals basis), cobalt(II) sulfate heptahydrate (CoSO_4_ × 7H_2_O, ≥99%), sodium borohydride (NaBH_4_, 99%), 2,2′-azino-bis (3-ethylbenzothiazoline-6-sulfonic acid (C_18_H_18_N_4_O_6_S_4_, ABTS, liquid) and D(+)-saccharose (C_12_H_22_O_11_; ≥99.5%) were purchased from Sigma-Aldrich Chemie GmbH (Steinheim, Germany). D-(+)-glucose monohydrate (C_6_H_12_O_6_ × H_2_O; ≥99.0%), diclofenac sodium (C_14_H_10_C_l2_NNaO_2_; ≥97.5%), acetylsalicylic acid (C_9_H_8_O_4_; ≥99%) and norfloxacin (C_16_H_18_FN_3_O_3_) were acquired from Alfa Aesar GmbH & Co KG (Ward Hill, MA, USA). D(-)-fructose (C_6_H_12_O_6_; ≥99.5%) was obtained from Merck KGaA (Darmstadt, Germany).

### 2.2. Preparation of Solutions

Amounts of 10 mM H_2_PtCl_6_, 0.1 M NaBH_4_, 10 mM CoSO_4_ and buffer solutions were prepared using ultra-high-quality (UHQ) water obtained by a DEMIWA rosa 5 water purification system (WATEK, Ledeč nad Sázavou, Czech Republic). A solution of 40.0 mg mL^−1^ GOx was prepared in a buffer solution containing 0.05 mM CH_3_COONa, 0.05 mM Na_2_HPO_4_ and 0.05 mM NaH_2_PO_4_. The buffer solution used for electrochemical studies was prepared by dissolving 0.05 mM CH_3_COONa, 0.05 mM Na_2_HPO_4_, 0.05 mM NaH_2_PO_4_ and 0.1 M KCl in UHQ water (AFBS) and adjusting its pH to 6.0. Standard solutions of 1.0 M H_2_O_2_, 0.05 M, 0.1 M and 1.0 M glucose, 1.0 M galactose, 1.0 M mannose, 1.0 M fructose, 1.0 M xylose, 1.0 M saccharose, 0.1 M ascorbic acid, 0.1 M uric acid, dopamine hydrochloride, 1.5 mM norfloxacin, 0.1 M acetaminophen, 0.1 M diclofenac sodium and 0.1 M acetylsalicylic acid were prepared in AFBS (pH 6.0). To ensure equilibrium between α- and β-anomeric forms, solutions of glucose and other saccharides were prepared at least 24 h before use for experiments. The GOx solution was aliquoted and stored at −22 °C temperature until experiments, while all other solutions were stored at +4 °C.

### 2.3. Synthesis and Characterization of PtCo Nanoparticles

The synthesis of bimetallic PtCo nanoparticles was carried out in two stages. First, 2 mL of 10 mM H_2_PtCl_6_ was mixed with 0.2 mL of 0.1 M NaBH_4_ and heated at 100 °C for 2 min in water bath. Then, 2 mL of 10 mM CoSO_4_ was added to the seed solution, followed by the addition of 0.5 mL of 0.1 M NaBH_4_. The resulting nanoparticles were collected by centrifugation at 8000× *g* for 30 min. The collected particles were washed twice with UHQ water and stored as an aqueous colloidal solution at +4 °C until use.

The PO-like activity of the synthesized PtCo nanoparticles was evaluated by their ability to oxidase ABTS in the presence of H_2_O_2_. The absorbance of the formed colored product was measured at a wavelength of 420 nm using a Shimadzu UV1650 PC spectrophotometer (Kyoto, Japan). The methodology is described in detail in a previously published article [20]. One unit (U) of PO-like activity is defined as the quantity of nanoparticles that decomposes 1 µM of H_2_O_2_ per minute under standard assay conditions. The measured PO-like activity of the PtCo nanoparticles was 0.32 U mL^−1^.

The morphology of the PtCo nanoparticles was investigated using a high-resolution field-emission scanning electron microscope (FE-SEM) SU-70 (Hitachi, Ibaraki, Japan), at magnifications of 50,000× and 100,000×. Samples for FE-SEM imaging were prepared by drop-casting 3 µL of PtCo nanoparticle suspension onto a silicon substrate using a micropipette and evaporating the solvent at ambient temperature.

### 2.4. Preparation of Working Electrodes

Graphite rods (99.999% purity, 3.0 mm diameter, 150 mm length) purchased from Sigma-Aldrich Chemie GmbH (Taufkirchen, Germany) were cut into pieces of approximately 4 cm length and hand-polished with fine (P120), very fine (P320), and finally ultra-fine (P2000) emery paper. The polished GRE were washed with ethanol and UHQ water, dried at ambient temperature, and then covered with a silicone tube to avoid contact of the lateral surface with the solution. When preparing the working electrode modified only with PtCo nanoparticles (GRE/PtCo/Nafion), 3 µL of PtCo solution was dropped onto the GRE surface with a micropipette, the solvent was evaporated at ambient temperature, then 3 µL of Nafion™ perfluorinated resin solution was dropped and the electrode was dried at ambient temperature for about 1 h. Meanwhile, when preparing a working electrode modified with both PtCo and GOx (GRE/PtCo/GOx/Nafion), a 40.0 mg mL^−1^ GOx solution was dropped onto the electrode surface before dropping 3 µL of Nafion™ perfluorinated resin solution. Under the optimized GRE/PtCo/GOx/Nafion preparation conditions, the electrode was modified by dropping 3 µL of PtCo solution twice, followed by dropping 3 µL of GOx solution twice, allowing the solvent to evaporate after each dropping step. Until experiments, the prepared GRE/PtCo/Nafion and GRE/PtCo/GOx/Nafion electrodes were stored in sealed test tubes above a drop of AFBS (pH 6.0) at +4 °C.

### 2.5. Electrochemical Measurements

A computerized potentiostat/galvanostat Autolab PGSTAT30 (Eco Chemie, Utrecht, The Netherlands) controlled by specialized NOVA1.9 software was used for all electrochemical studies. A conventional three-electrode electrochemical cell was used, consisting of GRE/PtCo/Nafion or GRE/PtCo/GOx/Nafion as the working electrode, an Ag/AgCl (3.0 M KCl) electrode as the reference electrode, and a platinum coil as the counter electrode. All measurements were performed in 5 mL of oxygenated AFBS (pH 6.0, unless a different pH was required), in a Faraday cage, and at ambient temperature. Amperometric measurements were carried out at a constant potential of −0.3 V while stirring the solution in the electrochemical cell with a magnetic stirrer at a speed of 400 rpm. Cyclic voltammetry experiments were performed by sweeping the potential at a scan rate of 0.1 V s^−1^ within a selected potential range.

### 2.6. Interpretation of Experimental Data

The electrochemical response of the biosensor is provided as a change in current (ΔI), calculated by subtracting the background current from the current measured after the addition of standard glucose solution to the electrochemical cell filled with AFBS (pH 6.0, unless otherwise required). Experiments were performed in triplicate unless otherwise stated, and obtained results are presented in the figures as mean values with error bars indicating standard deviation. The linear range of the dependence of the current response on glucose concentration was determined from the calibration curve, which was obtained by fitting a linear regression model (y = ax + b) to the experimental data. The slope (a), intercept (b), standard deviation of the response (σ), and coefficient of determination (R^2^) were also calculated based on this fitting. The LOD and the limit of quantification (LOQ) were calculated as 3.3 σ/a and 10 σ/a, respectively. The response time was determined as the duration (in seconds) required for the current to reach 90% of its steady-state value after a change in glucose concentration. Repeatability and reproducibility were evaluated by calculating the RSD of current responses obtained from five measurements of 2.0 mM glucose using the same GRE/PtCo/GOx/Nafion electrode and five independently prepared electrodes, respectively. The RSD, expressed as a percentage, was calculated by dividing the standard deviation by the mean of the measurements and multiplying by 100. The selectivity of the biosensor was assessed by measuring its current response to various saccharides. A glucose standard solution was initially added to the electrochemical cell to achieve a concentration of 2.0 mM. Once a steady-state current was reached, standard solutions of galactose, mannose, fructose, xylose, sucrose (each at 5.0 mM), and finally 2.0 mM of glucose were sequentially introduced, and the changes in current induced by these saccharides were recorded. Similarly, the influence of electroactive substances was evaluated. Standard solutions of glucose, ascorbic acid, dopamine hydrochloride, norfloxacin, acetaminophen, diclofenac sodium, acetylsalicylic acid, and glucose (again) were sequentially introduced into the electrochemical cell to reach concentrations of 2.0, 0.2, 0.1, 0.003, 0.2, 0.2, 0.2, and 2.0 mM, respectively. The operational stability of the biosensor was evaluated by repeatedly measuring the current response of three identically fabricated GRE/PtCo/GOx/Nafion electrodes to 2.0 mM of glucose at regular intervals over an 8-h period. To assess storage stability, the current response of three identically prepared GRE/PtCo/GOx/Nafion electrodes to 2.0 mM of glucose was measured at consistent time intervals over a period of 14 days. In both cases, the current responses were normalized and expressed as a percentage of the initial current measured either at the start of the 8-h test or on the first day of the 14-day test, and results were presented as the average of the three measurements. The accuracy of the biosensor was evaluated by analyzing commercial human serum. A 560 µL aliquot of serum with a known glucose concentration of 6.95 mM (determined using a FreeStyle Optium glucometer, ART16648 Rev. B 05/10) was added to an electrochemical cell containing 5.0 mL of AFBS buffer solution (pH 6.0). Following the measurement of the biosensor’s current response, the glucose concentration was determined using a linear equation derived from a calibration curve constructed from glucose standard solutions in AFBS (pH 6.0). Recovery (%) was calculated by dividing the determined glucose concentration by the added concentration and multiplying by 100.

## 3. Results and Discussion

### 3.1. Characterization of Morphological and PO-like Properties of PtCo Nanoparticles

**Figure 2:**
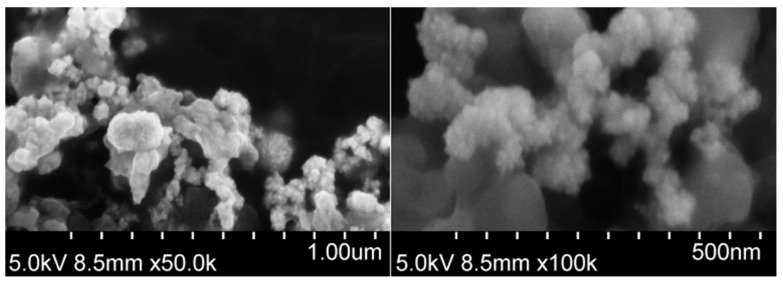
FE-SEM images of PtCo nanoparticles at 50,000× and 100,000× magnification.

As it is known that the catalytic activity of nanozymes is affected by their size and morphology [21,22], PtCo nanoparticles were characterized by FE-SEM. Figure 2 shows FE-SEM images of PtCo nanoparticles at 50,000× and 100,000× magnification. Although the exact size of each individual nanoparticle cannot be determined from the provided images, it is evident that they are smaller than 50 nm and tend to assemble into larger, three-dimensional, coral-like aggregates. This particle size and morphology suggest a high specific surface area. Previous studies on nanozymes have demonstrated that nanoparticles with smaller dimensions and specific morphologies, which contribute to increased surface area, generally exhibit enhanced catalytic activity [23]. For instance, the PO-like catalytic activity of Fe_3_O_4_ nanoparticles has been shown to increase with decreasing particle size [24]. Similarly, CoFe_2_O_4_ nanoparticles exhibit size- and shape-dependent PO-like catalytic activity, following the following order: spherical > near corner-grown cubic > starlike > near cubic > polyhedral [25].

**Figure 3:**
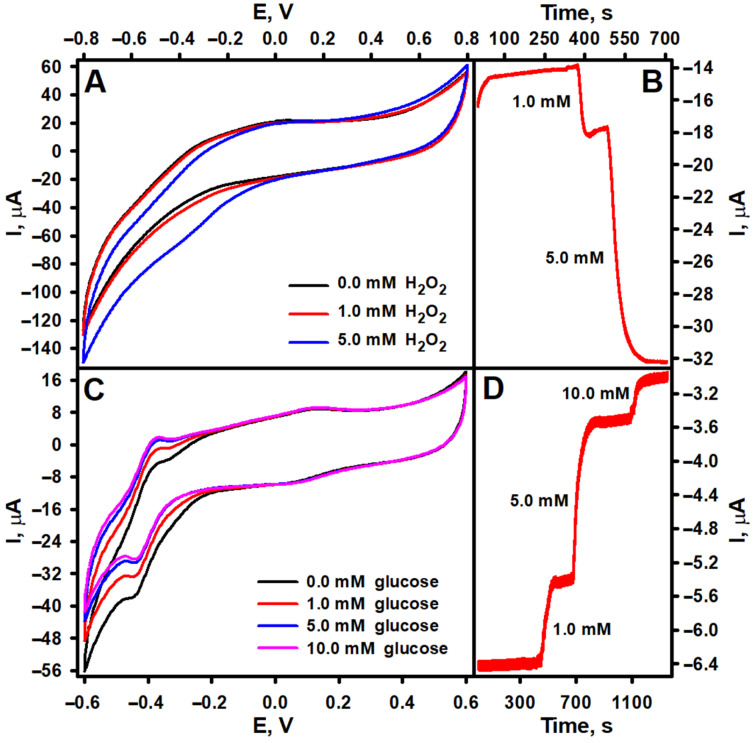
Cycling voltammograms and amperograms of GRE/PtCo/Nafion (**A**,**B**) and GRE/PtCo/GOx/Nafion (**C**,**D**) electrodes. GRE/PtCo/Nafion preparation conditions: 3 µL of 0.32 U mL^−1^ PtCo, 3 µL of Nafion™. GRE/PtCo/GOx/Nafion preparation conditions: 3 µL of 0.32 U mL^−1^ PtCo, 3 µL of 40.0 mg mL^−1^ GOx, 3 µL of Nafion™. Measurement conditions: AFBS (pH 6.0); −0.3 V applied potential vs. Ag/AgCl/KCl_3M_ (**B**,**D**); 0.1 V s^−1^ scan rate (**A**,**C**).

In this study, PtCo nanoparticles were utilized as functional nanomaterials for the modification of the biosensor’s working electrode, contributing to its electrocatalytic performance. Therefore, in addition to the spectrophotometric determination of the PO-like activity of the PtCo nanoparticles, which was 0.32 UmL^−1^, their ability to electrocatalyze the reduction of H_2_O_2_ was investigated by cyclic voltammetry and constant potential amperometry. As shown in Figure 3A, the reduction of H_2_O_2_ on the GRE/PtCo/Nafion electrode starts at a potential slightly more positive than 0 V, and the cathodic current increases as the applied potential becomes more negative. However, to enable selective and highly sensitive H_2_O_2_ reduction without interference from O_2_ reduction, a working potential of −0.3 V was selected for subsequent amperometric measurements. As shown in the amperogram provided in Figure 3B, a concentration-dependent cathodic current was recorded when H_2_O_2_ was added to the electrochemical cell. In order to confirm the catalytic effect of the PtCo nanoparticles, a control experiment using GRE without nanoparticles was performed. As can be seen from the cyclic voltammograms recorded for the bare GRE electrode, presented in Figure S1A, no change in the current was recorded after adding H_2_O_2_ to the electrochemical cell. In light of these findings, the GRE/PtCo/GOx/Nafion electrode was expected to generate a cathodic current in the presence of glucose. However, as can be seen from Figure 3C,D, a positive shift in the current generated by the electrode was observed when glucose was added to the electrochemical cell. A similar trend in current variation has been reported for some other amperometric biosensors based on oxidoreductases that produced H_2_O_2_ and peroxidase-mimicking nanozymes that catalyzed the reduction of this enzymatically generated H_2_O_2_. In most cases, the cause of this phenomenon has not been explained—for instance, in biosensors based on GOx and green-synthesized CuHCF micro/nanoflowers [20] and GOx combined with graphene quantum dot-supported silver nanoparticles [26], and in biosensors that use L-arginine oxidase with either NiPtPd nanoparticles or green-synthesized CuHCF micro/nanoflowers [16]. Meanwhile, in the case of a biosensor based on GOx and TiO_2_ nanoparticles encapsulated in a zeolitic imidazolate framework-8 (GOx@ZIF-8(TiO_2_)), the observed decrease in cathodic current with increasing glucose concentration was attributed to the exceptional tandem catalytic activity of GOx@ZIF-8(TiO_2_) for both glucose oxidation and H_2_O_2_ reduction [27]. According to the developers of the GOx and CuWO_4_ nanoparticle-based biosensor, this phenomenon may be associated with inhibition of the electrocatalytic reaction by the reaction catalyzed by enzyme molecules [28]. A similar rationale can be applied to the biosensor developed in this study. Several previous studies have demonstrated that PtCo nanoparticles exhibit high catalytic activity and stability for O_2_ reduction [29,30], which occurs on electrodes modified with these particles at more positive potentials than usual [31,32]. Typically, O_2_ reduction on carbon electrodes takes place at potentials below −0.3 to −0.6 V vs. Ag/AgCl [33,34]. However, though GRE electrodes modified solely with GOx (GRE/GOx/Nafion) generated only a minimal current response in the presence of glucose in O_2_-saturated buffer (Figure S1B,C, red line) when compared with GRE/PtCo/GOx/Nafion electrodes (Figure 3C,D), and no current response following deoxygenation with argon gas (Figure S1C, blue line), the incorporation of PtCo nanoparticles into the electrode architecture could likely alter its electrochemical properties and O_2_ reduction potential. In this situation, H_2_O_2_ reduction would occur simultaneously with O_2_ reduction. Upon addition of glucose, the GOx-catalyzed glucose oxidation reaction would consume dissolved O_2_, reducing its availability for electrochemical reduction at the electrode and resulting in a decrease in cathodic current. To confirm this assumption, the ability of GRE/PtCo/Nafion to catalyze the reduction of H_2_O_2_ and the ability of GRE/PtCo/GOx/Nafion to detect glucose in O_2_-free AFBS (pH 6.0) were investigated. Cyclic voltammograms of GRE/PtCo/Nafion electrodes in O_2_-saturated and deoxygenated solutions exhibited distinct differences (Figure S2A). Furthermore, a significantly higher reduction current was observed in the cyclic voltammogram of GRE/PtCo/Nafion under O_2_-saturated conditions. Corresponding amperometric measurements (Figure S2B) also showed that, in the absence of O_2_, the residual current was significantly lower, but the current resulting from H_2_O_2_ reduction was very similar in magnitude to the current recorded in O_2_-saturated solution. Meanwhile, GRE/PtCo/GOx/Nafion in deoxygenated solution showed a clear decrease in the redox peak current (Figure S3A) compared with the O_2_-saturated solution (Figure 3C). Furthermore, no current response was observed in the presence of glucose under O_2_-free conditions during amperometric measurements, highlighting the necessity of O_2_ for enzymatic activity (Figure S3B). However, despite the potentially more complex mechanism of action of the biosensor than that shown in Figure 1, involving both enzymatic O_2_ consumption and an electrocatalytic H_2_O_2_ reduction process, the current it generated under O_2_-saturated conditions was clearly dependent on glucose concentration (Figure 3D and Figure S3B). This demonstrates the potential of PtCo nanoparticles for the development of electrochemical biosensors.

### 3.2. Optimization of GRE/PtCo/GOx/Nafion Composition and Buffer pH

**Figure 4:**
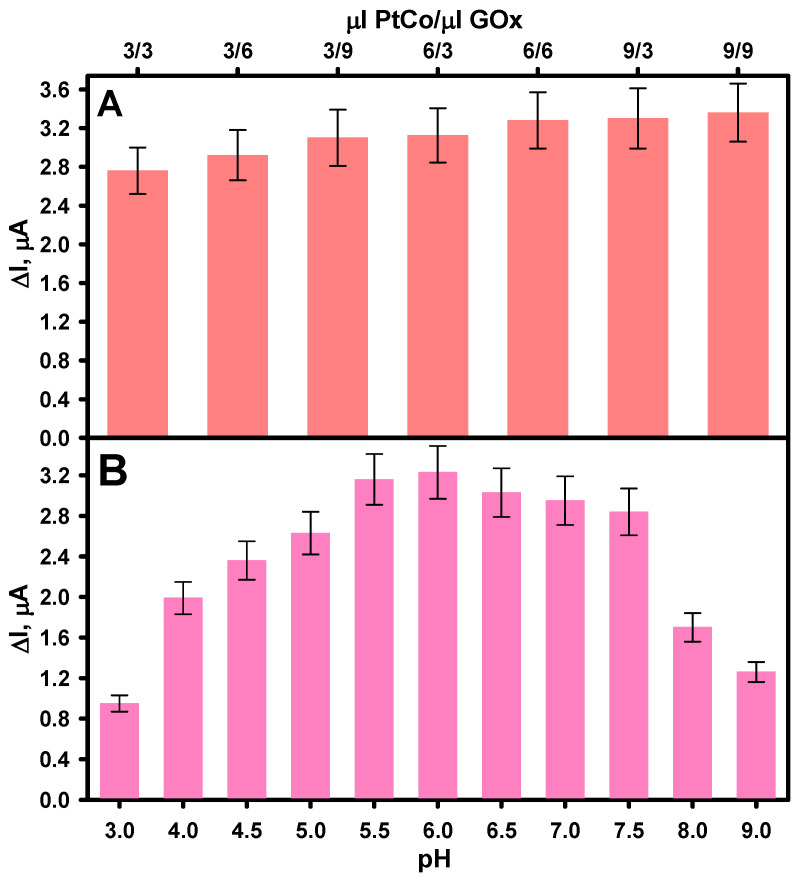
Influence of PtCo and GOx loading (**A**) and AFBS buffer solution pH (**B**) on the current response of GRE/PtCo/GOx/Nafion electrodes. GRE/PtCo/GOx/Nafion preparation conditions: 6 µL of 0.32 U mL^−1^ PtCo, 6 µL of 40.0 mg mL^−1^ GOx, 3 µL of Nafion™ (**B**). Measurement conditions: AFBS (pH 6.0) (**A**); 2.0 mM of glucose, −0.3 V applied potential vs. Ag/AgCl/KCl_3M_.

The pH of the buffer solution and the quantities of PtCo and GOx on the working electrode surface were experimentally optimized to achieve the best possible biosensor performance. According to the proposed simplified illustration of the operating principle of the developed amperometric enzyme–nanozyme biosensor, shown in Figure 1, its operation relies on glucose oxidation catalyzed by immobilized GOx in the presence of dissolved O_2_, alongside the PtCo nanoparticles’ ability to catalyze the reduction of enzymatically generated H_2_O_2_. Therefore, both PtCo and GOx can affect the magnitude of the current generated by the biosensor. Figure 4A shows the relationship between the biosensor’s response current and the loading amounts of PtCo and GOx at a fixed glucose concentration (2.0 mM). An enhancement in the response current was observed with increasing loadings of both enzyme and nanoparticles on the surface of the GRE electrode. This improvement with increasing GOx loading is attributed to the formation of a higher amount of H_2_O_2_, which in turn produces a higher electrochemical signal [35]. Similarly, higher PtCo loading likely contributes to improved catalytic reduction of H_2_O_2_ and an increased effective electrode surface area due to the nanoparticles’ high surface-area-to-volume ratio [36]. However, when the loading of both GOx and PtCo exceeded 6 µL, the response current increased only very slightly, indicating saturation of the catalytic sites or mass transfer limitations [37]. Consequently, these volumes (6 µL of 40.0 mg mL^−1^ GOx and 6 µL of 0.32 U mL^−1^ PtCo) were selected as optimal for loading both the enzyme and the nanozyme in subsequent experiments.

The pH of the buffer solution is one of the most important factors affecting the performance of enzymatic biosensors, as it directly affects enzyme tertiary structure and, consequently, catalytic activity. At pH values below 4, GOx undergoes denaturation, leading to significant loss of activity. Meanwhile, at pH values above 8, the GOx activity decreases due to insufficient proton availability for catalysis [38]. Numerous studies have shown that free GOx exhibits its highest catalytic activity within the pH range of 5.5 to 7.0, with optimal stability and reactivity observed near neutral pH [39,40,41]. Furthermore, it has been reported that the properties of metal nanoparticle-based nanozymes are also pH-dependent, with PO-like activity generally predominating at acidic pH [42]. This is because their catalytic performance is determined by the pH-dependent H_2_O_2_ adsorption and decomposition behavior on their surface, which affects the reaction pathway and overall H_2_O_2_ reduction efficiency [43]. Therefore, the influence of AFBS buffer solution pH on the current response to glucose generated by the biosensor was investigated. As shown in Figure 4B, the current response was clearly dependent on pH in the range of 3.0–9.0. The highest current was recorded at pH 6.0, indicating optimal activity of both GOx and PtCo nanoparticles. Therefore, an AFBS buffer solution pH 6.0 was selected as optimal for biosensor performance and further glucose detection.

### 3.3. Evaluation of the Analytical Performance of the Biosensor

**Figure 5:**
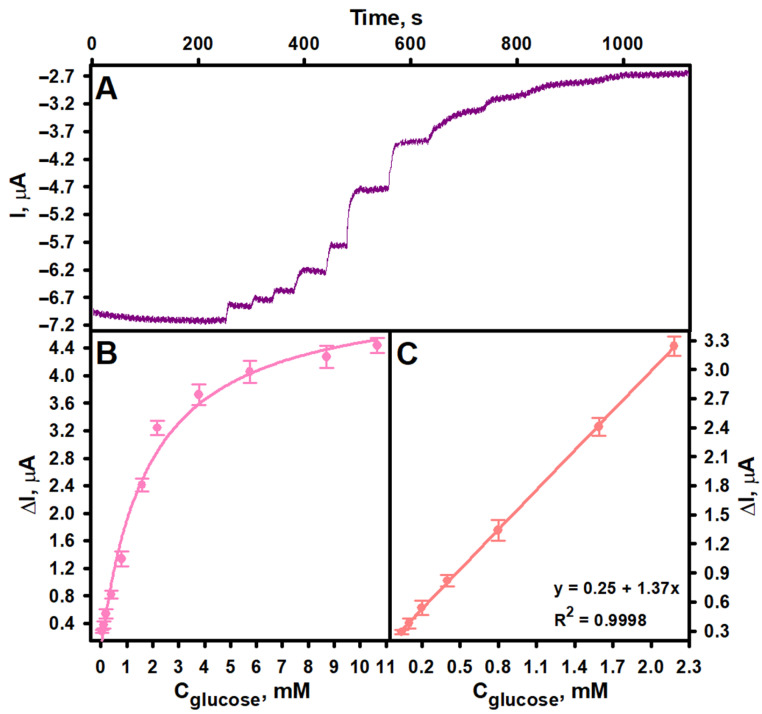
Dependence of the biosensor current response on glucose concentration (**A**,**B**) and calibration curve (**C**). GRE/PtCo/GOx/Nafion preparation conditions: 6 µL of 0.32 U mL^−1^ PtCo and 6 µL of 40.0 mg mL^−1^ GOx, 3 µL of Nafion™. Measurement conditions: AFBS (pH 6.0); −0.3 V applied potential vs. Ag/AgCl/KCl_3M_; 0.04, 0.10, 0.20, 0.40, 0.80, 1.59, 2.18, 3.78, 5.75, 8.70 and 10.66 mM of glucose.

To evaluate the performance of the developed biosensor, the current response of the GRE/PtCo/GOx/Nafion electrode, prepared under optimized conditions, was investigated at a constant potential of −0.3 V by sequentially adding a glucose standard solution to an electrochemical cell containing oxygenated AFBS (pH 6.0), thereby gradually increasing the concentration of the analyte. As shown in Figure 5A,B, the current response increased gradually with increasing glucose concentration and reached a steady state when the concentration in the electrochemical cell exceeded approximately 10 mM. Figure 5C presents the calibration curve of the biosensor, demonstrating a linear response to glucose in the concentration range of 0.04 to 2.18 mM (R^2^ = 0.9998). The LOD was calculated to be 0.02 mM, while the LOQ was 0.064 mM. The detection sensitivity was determined to be 1.37 ± 0.01 μA mM^−1^ (19.38 μA mM^−1^ cm^−2^). The response time was dependent on glucose concentration but remained below 10 s throughout the entire linear range. The LOD and other analytical performance parameters of the developed biosensor were comparable to, and in some cases exceeded, those reported for previously published amperometric biosensors based on PO-like nanozymes. This is evident from the biosensors comparison presented in Table 1. This can be attributed to the good catalytic activity of both GOx and PtCo, as well as their synergistic action under the selected analytical conditions. Furthermore, it is important to note that, despite the fact that PtCo nanoparticles with PO-like activity have previously been employed in colorimetric analytical systems [44,45], this study is the first to utilize them in the development of an amperometric biosensor.

Reproducibility and repeatability of the analytical signal were investigated and evaluated, as these are among the most critical analytical parameters influencing the reliability and practical applicability of biosensors [47]. The repeatability of the biosensor was assessed by recording its current response to an identical glucose concentration in five consecutive measurements using the same GRE/PtCo/GOx/Nafion electrode. As presented in Table 2, the RSD calculated for the data obtained in the repeatability study was 4.90%, which indicates the good repeatability of the developed biosensor. The reproducibility of the biosensor was also evaluated by recording the current response to identical glucose concentrations using five independently but identically prepared GRE/PtCo/GOx/Nafion electrodes. The RSD calculated from this study was 8.90%, indicating that the biosensor exhibited satisfactory reproducibility. The higher variability observed in the reproducibility study, compared with the repeatability study, is most likely due to minor inconsistencies in the fabrication of the GRE/PtCo/GOx/Nafion electrodes, particularly variations in the amount of PtCo and/or GOx deposited on the electrode surface.

**Figure 6:**
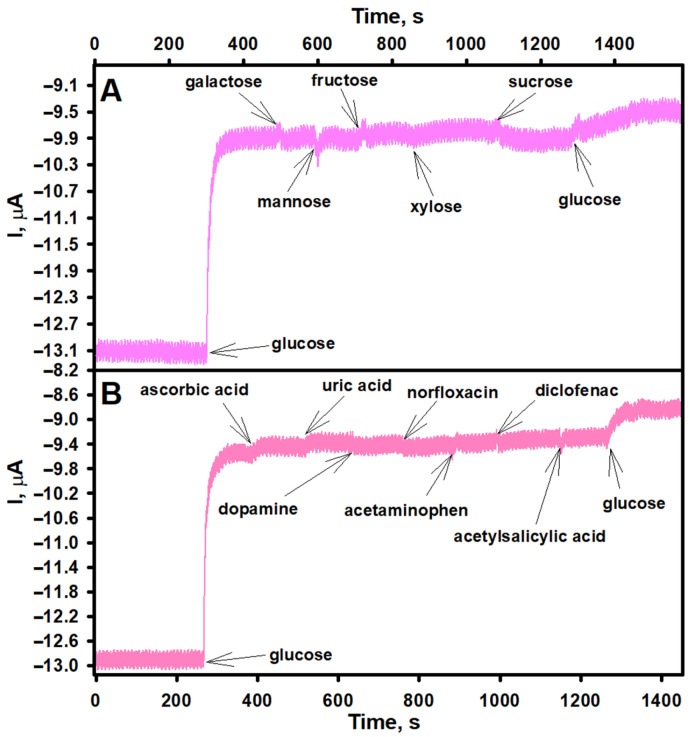
Current response of the biosensor to glucose and other saccharides (**A**) or some interfering substances (**B**). GRE/PtCo/GOx/Nafion preparation conditions: 6 µL of 0.32 U mL^−1^ PtCo and 6 µL of 40.0 mg mL^−1^ GOx, 3 µL Nafion™. Measurement conditions: AFBS (pH 6.0); −0.3 V applied potential vs. Ag/AgCl/KCl_3M_; 2.0 mM of glucose and 5.0 mM of other saccharides (**A**); 2.0 mM of glucose, 0.2 mM of ascorbic acid, 0.5 mM of uric acid; 0.1 mM of dopamine, 3.0 µM of norfloxacin, 0.2 mM of acetaminofen, 0.2 mM of diclofenac, and 0.2 mM of acetylsalicylic acid (**B**).

Although enzymes are inherently highly selective toward their target analytes, the selectivity of a newly developed biosensor must still be thoroughly evaluated. In the case of the GRE/PtCo/GOx/Nafion working electrode, the current response of the biosensor may be influenced not only by the enzymatic activity of GOx but also by the electrocatalytic properties of the PtCo nanoparticles. Notably, nanoporous Pt–Co alloys and nanostructured composites have been reported to play a significant role in the electrocatalytic oxidation of glucose in non-enzymatic sensors [48,49]. Other saccharides, including fructose and sucrose, can also undergo electrooxidation with high efficiency on Pt-based surfaces [50]. Conversely, several studies on non-enzymatic H_2_O_2_ sensors indicate that glucose has no effect or only a negligible effect on the current response of these sensors [51,52]. Therefore, to ensure the specificity of the developed biosensor, its selectivity toward glucose was also investigated. As shown in Figure 6A, none of the tested saccharides produced a noticeable change in the current response of the biosensor, demonstrating its good selectivity for glucose under the selected analysis conditions.

In addition to glucose, real biological samples may contain various electroactive substances that, under the applied working potential, can undergo oxidation or reduction on the surface of the working electrode, thereby potentially interfering with the current response of the biosensor to glucose and thus affect the current signal generated by the biosensor. For glucose biosensors, common interfering substances include uric acid, ascorbic acid, and certain pharmaceuticals [53]. Therefore, the anti-interference capability of the developed biosensor was evaluated by comparing its current response to glucose with those caused by several potentially interfering substances, including ascorbic acid, dopamine, norfloxacin, acetaminophen, diclofenac, and acetylsalicylic acid. As shown in Figure 6B, a pronounced increase in current was observed after the addition of glucose. In contrast, the interfering substances caused negligible current changes, despite being tested at concentrations significantly higher than their typical physiological levels. For instance, the physiological concentration of ascorbic acid rarely exceeds 0.1 mM [54]. However, a second addition of glucose resulted in another clear increase in current. These findings demonstrate the excellent anti-interference performance of the developed biosensor. This can be attributed to the presence of the Nafion layer on the GRE/PtCo/GOx/Nafion working electrode. Nafion is often used as a composite film to improve the anti-interference capability of biosensors, as its negatively charged sulfonate groups effectively repel negatively charged interferents from the electrode surface [35].

**Figure 7:**
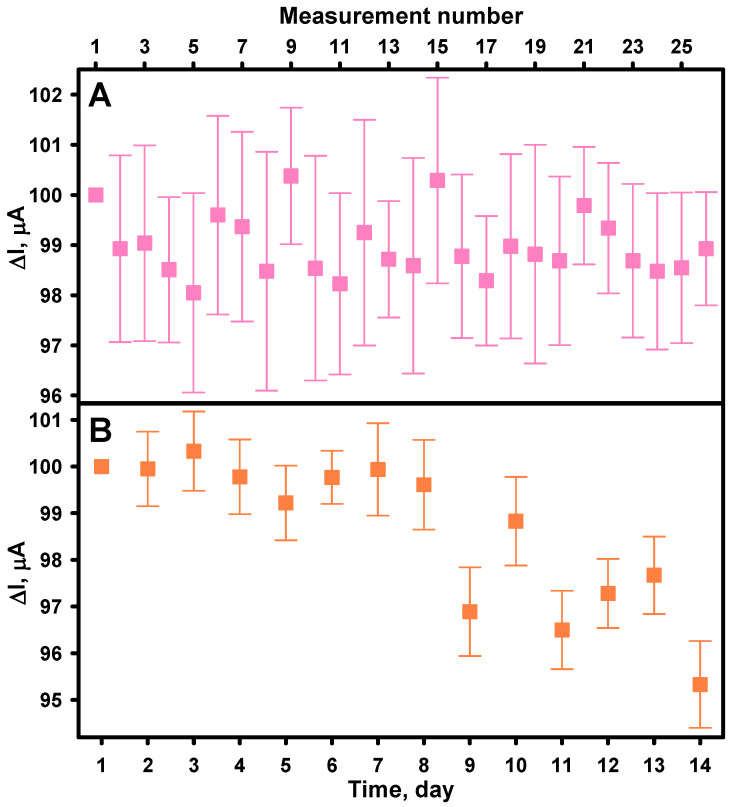
Operational (**A**) and storage (**B**) stability of the biosensor. GRE/PtCo/GOx/Nafion preparation conditions: 6 µL of 0.32 U mL^−1^ PtCo, 6 µL of 40.0 mg mL^−1^ GOx, and 3 µL Nafion™. Measurement conditions: AFBS (pH 6.0); −0.3 V applied potential vs. Ag/AgCl/KCl_3M_; 2.0 mM of glucose.

Biosensor stability, encompassing both operational and long-term stability, is a very important performance parameter of biosensors, especially when they are used for continuous or repeated clinical, environmental, or industrial applications. Signal attenuation over time due to enzyme denaturation, electrode fouling, or degradation of other materials used in its construction can affect the accuracy and reliability of the biosensor, potentially leading to false analytical results [55,56]. Operational stability refers to the ability of a biosensor to maintain consistent performance during continuous or repeated use, while long-term stability concerns the shelf-life and storage stability of the device. The operational stability of the biosensor was evaluated by repeatedly measuring the current response of the GRE/PtCo/GOx/Nafion electrode to 2.0 mM glucose at regular intervals over an 8-h period. As shown in Figure 7A, the biosensor maintained 98.93% of the initial response after 26 cycles of glucose detection. To evaluate the storage stability of the developed biosensor, its current response to 2 mM glucose was measured once daily over a 14-day period. As shown in Figure 7B, the biosensor retained 95.33% of its initial response, indicating minimal performance loss. These results suggests that both GOx and PtCo nanoparticles maintained their catalytic activity under the given storage and testing conditions, confirming the suitability of the biosensor for continuous or repeated applications over the studied timeframe.

### 3.4. Analysis of Human Serum Samples

The practical applicability of the developed biosensor was evaluated by determining the glucose concentration in a commercial human serum. To this end, a 560 µL aliquot of serum with a known glucose concentration of 6.95 mM was introduced into an electrochemical cell containing 5.0 mL of AFBS buffer solution (pH 6.0), yielding a final glucose concentration of 0.70 mM (dilution factor: 9.93). This concentration was deliberately selected to fall within the linear detection range of the biosensor. The measurement was performed in triplicate under identical conditions. Glucose concentration was determined using a previously established calibration curve constructed from glucose standard solutions prepared in AFBS (pH 6.0). As shown in Table 3, the average recovery was calculated to be 100.95%, with the RSD of less than 6%. These results demonstrate the high accuracy and precision of the developed biosensor and confirm its applicability for glucose determination in complex biological matrices.

## 4. Conclusions

In this study, a novel amperometric enzyme–nanozyme biosensor for glucose detection was developed and characterized. To develop the biosensor, GRE was sequentially modified with PtCo nanoparticles, GOx, and Nafion™ perfluorinated resin using a layer-by-layer deposition approach. The PtCo nanoparticles, deposited onto the GRE surface, exhibited PO-like catalytic activity and effectively electrocatalyzed the reduction of H_2_O_2_. Thus, the developed biosensor exploited the enzymatic oxidation of glucose by immobilized GOx in the presence of dissolved O_2_, along with the PtCo nanozyme-catalyzed reduction of enzymatically produced H_2_O_2_. This synergistic action of the native enzyme and the nanozyme ensured a high sensitivity to glucose, with low limits of glucose detection and quantification. In addition to this analytical performance, the biosensor exhibited excellent repeatability and operational and storage stability. It also showed remarkable selectivity toward glucose and strong resistance to interference from common electroactive species. Furthermore, the biosensor achieved accurate glucose quantification in commercial human serum samples, demonstrating its applicability in complex biological matrices. The simplicity and efficiency of the biosensor design suggest that it can be adapted for the detection of other clinically or environmentally relevant analytes.

## Figures and Tables

**Table 1:** Comparison of the analytical parameters of the developed biosensor and some previously reported biosensors based on PO-like nanozymes.

Working Electrode	Linear Range, mM	LOD, mM	LOQ, mM	Sensitivity, μA mM^−1^ cm^−2^	Response Time, s	Stability, % From Initial	Ref.
GRE/PtCo/GOx/Nafion	0.04–2.18	0.021	0.064	19.38	<10	95.33% after 14 days	this work
GRE/CuHCF/GOx/Nafion	up to 0.50	–	–	32.2	–	–	[20]
Pt/GOx/Fe_3_O_4_/Cs/Nafion	0.006–2.2	0.006	–	11.54	–	84% after 1 month	[35]
LSG/CeO_2_-MoS_2_/AuNPs/LOx	0.1–1 1–50	0.052	–	25.58 2.35	–	96.6% after 25 days	[46]
GCE/CuWO_4_/GOx/Nafion	0.005–1.8	0.0015	–	28.02	–	–	[28]
GCE/NiFe_2_/OMC/GOx/Nafion	0.0486–12.5	0.0027	–	6.9	–	93% after 4 weeks	[19]
GCE/GOx@ZIF-8(TiO_2_)	0.00008	–	–	–	<5	–	[27]
Au/RGO/PtPd/GOx	2–12	0.001	–	24	<5	–	[18]

Cs—chitosan; LSG—laser-scribed graphene; LOx—lactate oxidase; AuNPs—gold nanoparticles; OMC—ordered mesoporous carbon; RGO—reduced graphene oxide.

**Table 2:** Data obtained during the study of repeatability and reproducibility.

Study	ΔI, µA	Average of ΔI, µA	STDEV	RSD, % (n = 5)
Repeatability	2.61	2.81	0.14	4.90
	2.90			
	2.82			
	2.96			
	2.74			
Reproducibility	3.63	3.19	0.28	8.90
	3.15			
	3.27			
	2.96			
	2.93			

**Table 3:** Data obtained from the determination of glucose concentration in human serum.

Added Glucose, mM	Detected Glucose, mM	Recovery, %	Average, %	RSD, % (n = 3)
0.70	0.66	94.29	100.95	5.89
0.70	0.74	105.71		
0.70	0.72	102.86		

## Data Availability

The data presented in this study are available on request from the first author.

## Supplementary Materials

The following supporting information can be downloaded at https://www.mdpi.com/article/10.3390/bios15080545/s1, Figure S1: Cycling voltammograms of bare GRE (A) and GRE/GOx/Nafion (B) electrodes and amperogram of a GRE/GOx/Nafion electrode (C) in oxygen-saturated solution (red line) and deoxygenated buffer solution (blue line); Figure S2: Cycling voltammograms of a GRE/PtCo/Nafion electrode in oxygen-saturated and deoxygenated buffer solution (A). Amperograms of a GRE/PtCo/Nafion electrode in oxygen-saturated (blue line) and deoxygenated (red line) buffer solutions (B); Figure S3: Cycling voltammograms of a GRE/PtCo/GOx/Nafion electrode in deoxygenated buffer solution (A). Amperograms of a GRE/PtCo/GOx/Nafion electrode in oxygen-saturated (blue line) and deoxygenated (red line) buffer solutions (B).

## Abbreviations

GOx	Glucose oxidase
PtCo	platinum cobalt nanoparticles
RSD	Relative standard deviation
PO	Peroxidase
PO-MNZs	Metal nanoparticle-based nanozymes with peroxidase-like properties
LOD	Limit of detection
GCE	Glassy carbon electrode
GRE	Graphite rod electrode
UHQ	Ultra-high quality
ABTS	2,2′-azino-bis (3-ethylbenzothiazoline-6-sulfonic acid
AFBS	Buffer solution prepared by dissolving CH_3_COONa, Na_2_HPO_4_, NaH_2_PO_4_ and KCl in ultra-high-quality water
FE-SEM	Field-emission scanning electron microscope
GRE/PtCo/Nafion	Graphite rod electrode modified with PtCo nanoparticles
GRE/PtCo/GOx/Nafion	Graphite rod electrode modified with PtCo nanoparticles and glucose oxidase
R^2^	Coefficient of determination
LOQ	Limit of quantification
GOx@ZIF-8(TiO_2_)	Glucose oxidase and TiO_2_ nanoparticles encapsulated in a zeolitic imidazolate framework-8
GRE/GOx/Nafion	Graphite rod electrode modified with glucose oxidase
Cs	Chitosan
LSG	Laser-scribed graphene
LOx	Lactate oxidase
AuNPs	Gold nanoparticles
OMC	Ordered mesoporous carbon
RGO	Reduced graphene oxide
STDEV	Standard deviation
