# Supplementary material for: An Amperometric Enzyme–Nanozyme Biosensor for Glucose Detection
Source: Biosensors (Basel). 2025 Aug 19;15(8):545. doi: 10.3390/bios15080545 (PMC12384605; doi:10.3390/bios15080545)
Supplement: Supplementary file 1 [file biosensors-15-00545-s001.zip › biosensors-3778912-supplementary.pdf]

Article

An Amperometric Enzyme–Nanozyme Biosensor for Glucose Detection

Asta Kausaite-Minkstimiene [1,*], Aiste Krikstaponyte [1], Nataliya Stasyuk [2], Galina Gayda [2] and Almira Ramanaviciene [1,*]

[1] NanoTechnas – Center of Nanotechnology and Materials Science, Faculty of Chemistry and Geosciences, Vilnius University, Naugarduko st. 24, LT-03225, Vilnius, Lithuania; asta.kausaite@chf.vu.lt (A.K.-M.); aiste.krikstaponyte@chgf.vu.lt (A.K.); almira.ramanaviciene@chf.vu.lt (A.R.)

[2] Department of Analytical Biotechnology, Institute of Cell Biology National Academy of Sciences of Ukraine (ICB NASU), Dragomanova str. 14/16, 79005 Lviv, Ukraine; stasukne@nas.gov.ua (N.S.); galina.gayda@nas.gov.ua (G.G.)

* Correspondence: asta.kausaite@chf.vu.lt; almira.ramanaviciene@chf.vu.lt; Tel.: +370 5 219 3115

Supplementary Materials

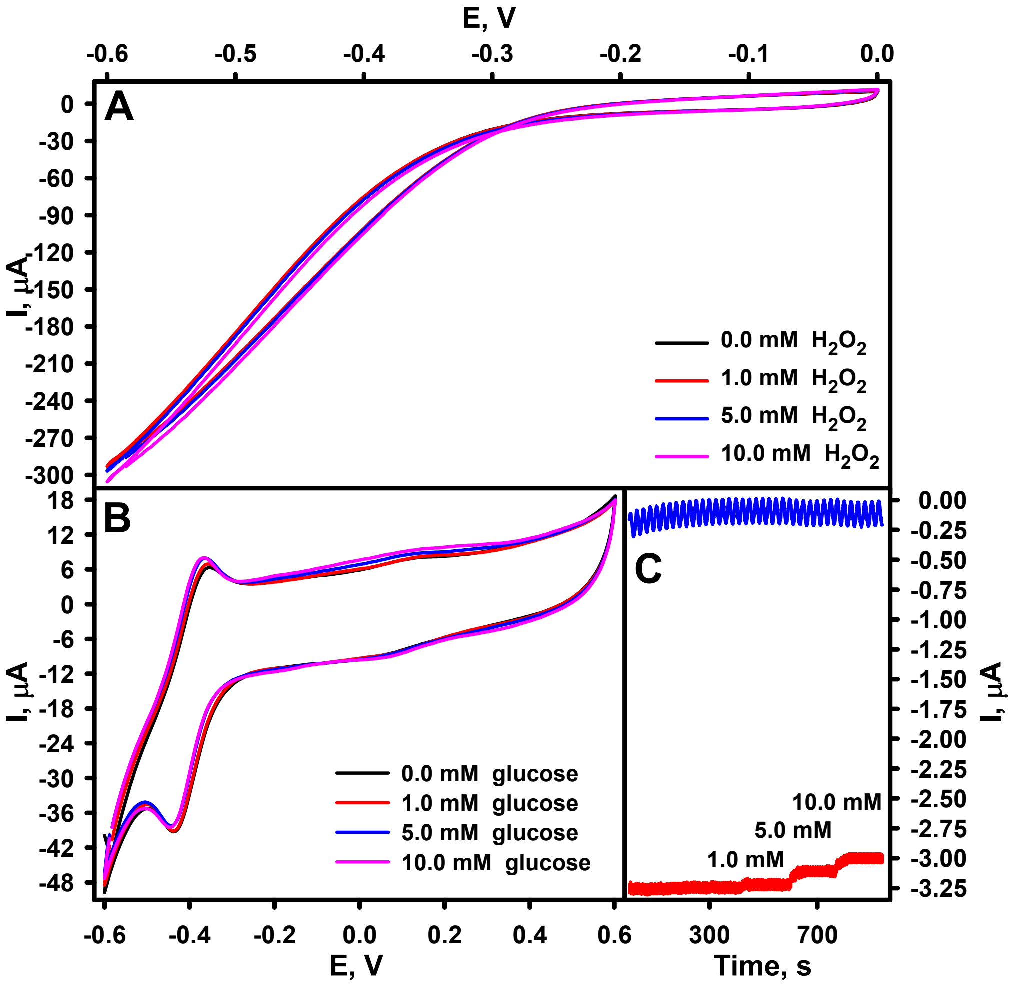

Figure S1. Cycling voltammograms of bare GRE (**A**) and GRE/GOx/Nafion (**B**) electrodes and amperogram of a GRE/GOx/Nafion electrode (**C**) in oxygen-saturated solution (red line) and deoxygenated buffer solution (blue line). GRE/GOx/Nafion preparation conditions: 3 μL of

40.0 mg mL^{-1} GOx, 3 μL of Nafion™. Measurement conditions: AFBS (pH 6.0); –0.3 V applied potential vs. Ag/AgCl/KCl$_{3M}$ (**C**); 0.1 V s^{-1} scan rate (**A**,**B**).

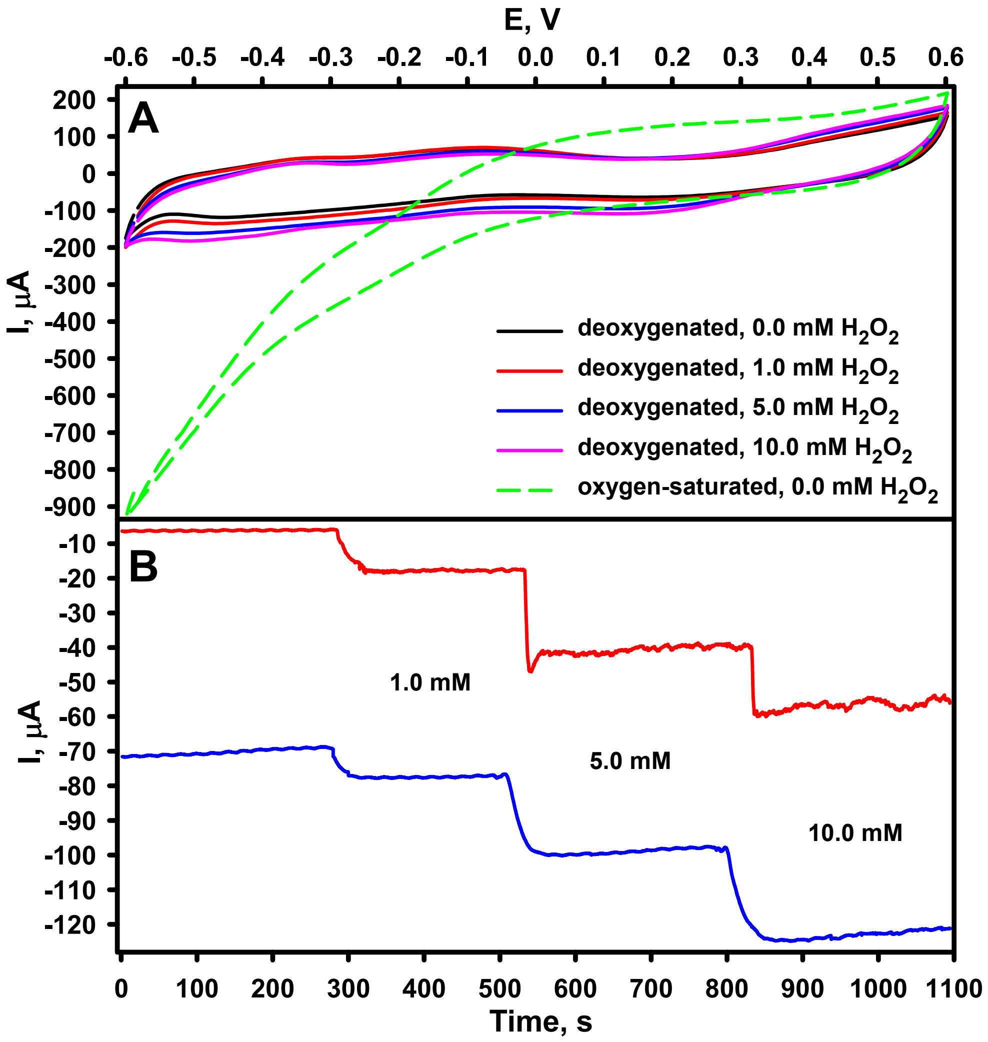

Figure S2. Cycling voltammograms of GRE/PtCo/Nafion electrode in oxygen saturated and deoxygenated buffer solution (**A**). Amperograms of GRE/PtCo/Nafion electrode in oxygen-saturated (blue line) and deoxygenated (red line) buffer solutions (**B**). GRE/PtCo/Nafion preparation conditions: 6 μL of 0.32 U mL^{-1} PtCo, 3 μL of Nafion™. Measurement conditions: AFBS (pH 6.0); –0.3 V applied potential vs. Ag/AgCl/KCl$_{3M}$ (**B**); 0.1 V s^{-1} scan rate (**A**).

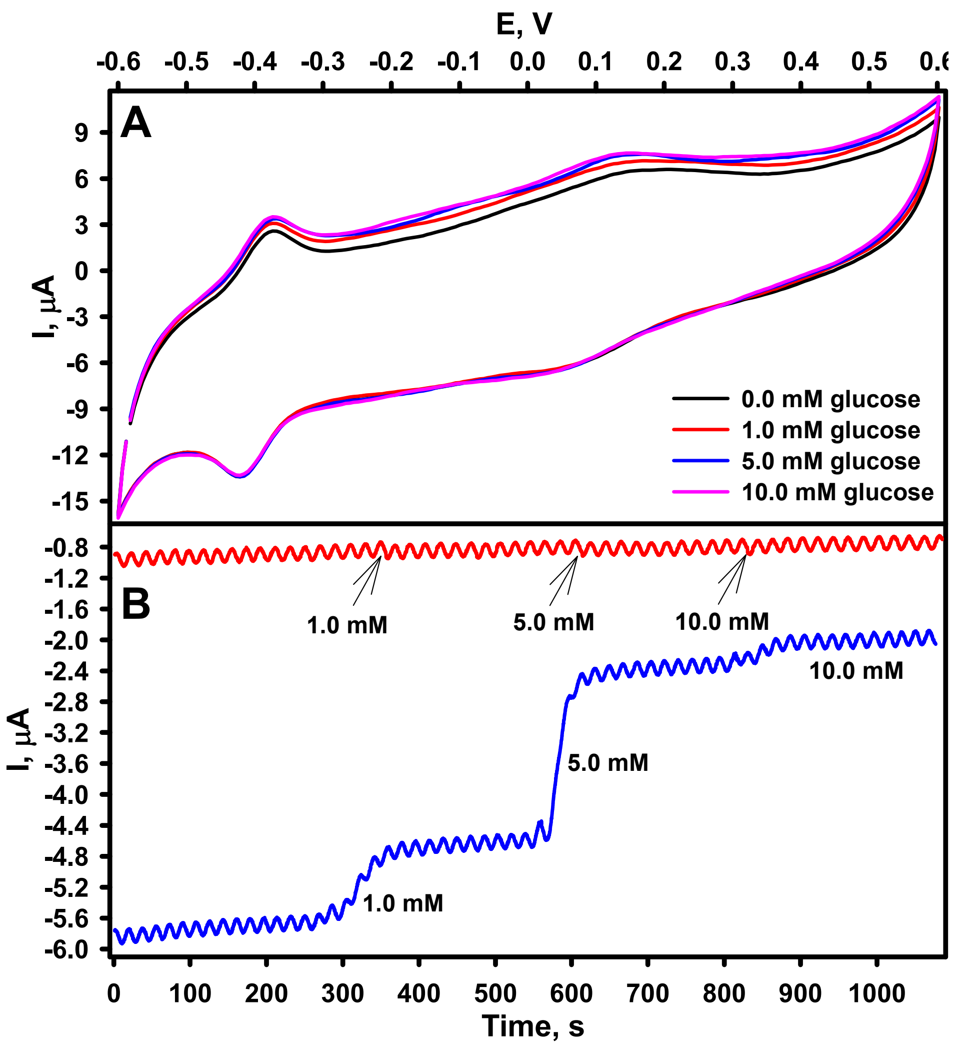

Figure S3. Cycling voltammograms of GRE/PtCo/GOx/Nafion electrode in deoxygenated buffer solution (**A**). Amperograms of GRE/PtCo/GOx/Nafion electrode in oxygen-saturated (blue line) and deoxygenated (red line) buffer solutions (**B**). GRE/PtCo/GOx/Nafion preparation conditions: 6 μL of 0.32 U mL^{-1} PtCo, 6 μL of 40.0 mg mL^{-1} GOx, 3 μL of Nafion™. Measurement conditions: AFBS (pH 6.0); –0.3 V applied potential vs. Ag/AgCl/KCl_{3M} (**B**); 0.1 V s^{-1} scan rate (**A**).
